# Multimodal Imaging in Indolent Nonprogressive Multifocal Choroidal Lesions: A Case Report

**DOI:** 10.7759/cureus.107371

**Published:** 2026-04-19

**Authors:** Ryota Ando, Shuhei Tomita, Masayo Kimura, Miho Nozaki

**Affiliations:** 1 Department of Ophthalmology, Nagoya City University East Medical Center, Nagoya, JPN

**Keywords:** choroidal diseases, differential diagnosis, ｗide-field octa, indolent nonprogressive multifocal choroidal lesions, optical coherence tomography angiography, ｍultimodal imaging

## Abstract

Indolent nonprogressive multifocal choroidal lesions are rare benign lymphoid infiltrative disorders confined to the choroid. A 53-year-old asymptomatic man was incidentally found to have a hyperdense lesion adjacent to the left optic nerve on head computed tomography. Fundus examination revealed multiple deep yellow-orange choroidal lesions bilaterally. Fluorescein angiography demonstrated late-phase staining in some lesions, whereas indocyanine green angiography showed more extensive hypofluorescent areas than were visible on color fundus imaging. Swept-source optical coherence tomography (OCT) demonstrated multiple hyporeflective cavernous lesions within the choroid. Wide-field OCT angiography revealed patchy choroidal flow signal alterations with mild attenuation in the overlying choriocapillaris, without abnormalities in the retinal capillary plexuses. Wide-field multimodal imaging facilitated noninvasive characterization and longitudinal assessment of this rare condition, which is particularly important given the lack of established diagnostic criteria and the need for accurate differentiation from other choroidal diseases.

## Introduction

Indolent nonprogressive multifocal choroidal lesions are rare benign lymphoid infiltrative disorders confined to the choroid [1]. Here, we report a case evaluated using multimodal imaging, highlighting its utility in visualizing lesion distribution and retinochoroidal vascular characteristics. These lesions present as multiple white-to-yellow patchy lesions and are typically asymptomatic, with minimal effect on visual function. Because of their rarity and the absence of established diagnostic criteria, careful differentiation from infectious, inflammatory, and neoplastic choroidal diseases―such as syphilis, tuberculosis, birdshot chorioretinopathy, acute posterior multifocal placoid pigment epitheliopathy (APMPPE), multiple evanescent white dot syndrome (MEWDS), sarcoidosis, and choroidal lymphoma―is required. Biopsy is often impractical owing to lesion location, and diagnosis generally relies on multimodal imaging and exclusion of other conditions. Previous reports have described characteristic imaging findings of indolent nonprogressive multifocal choroidal lesions. On structural optical coherence tomography (OCT), these lesions appear as multiple hyporeflective areas within the choroid with preservation of the overlying retinal structures [1-4]. Indocyanine green angiography (ICGA) typically shows more extensive hypofluorescent lesions than those visible on color fundus imaging, whereas fluorescein angiography (FA) may demonstrate minimal or late-phase staining [1-4]. Fundus autofluorescence is usually unremarkable, reflecting limited involvement of the retinal pigment epithelium [3,4]. However, detailed evaluation using wide-field imaging modalities has not been well characterized. Wide-field OCT and optical coherence tomography angiography (OCTA), as part of multimodal imaging techniques, enable noninvasive visualization of lesion distribution and retinochoroidal vascular characteristics compared with conventional imaging modalities, thereby improving the accuracy of detection, differentiation, and longitudinal monitoring of choroidal diseases. In this study, we performed multimodal imaging, including wide-field OCTA and wide-field en face OCT, and report their utility in the noninvasive assessment and follow-up of this rare condition.

## Case presentation

**Figure 1:**
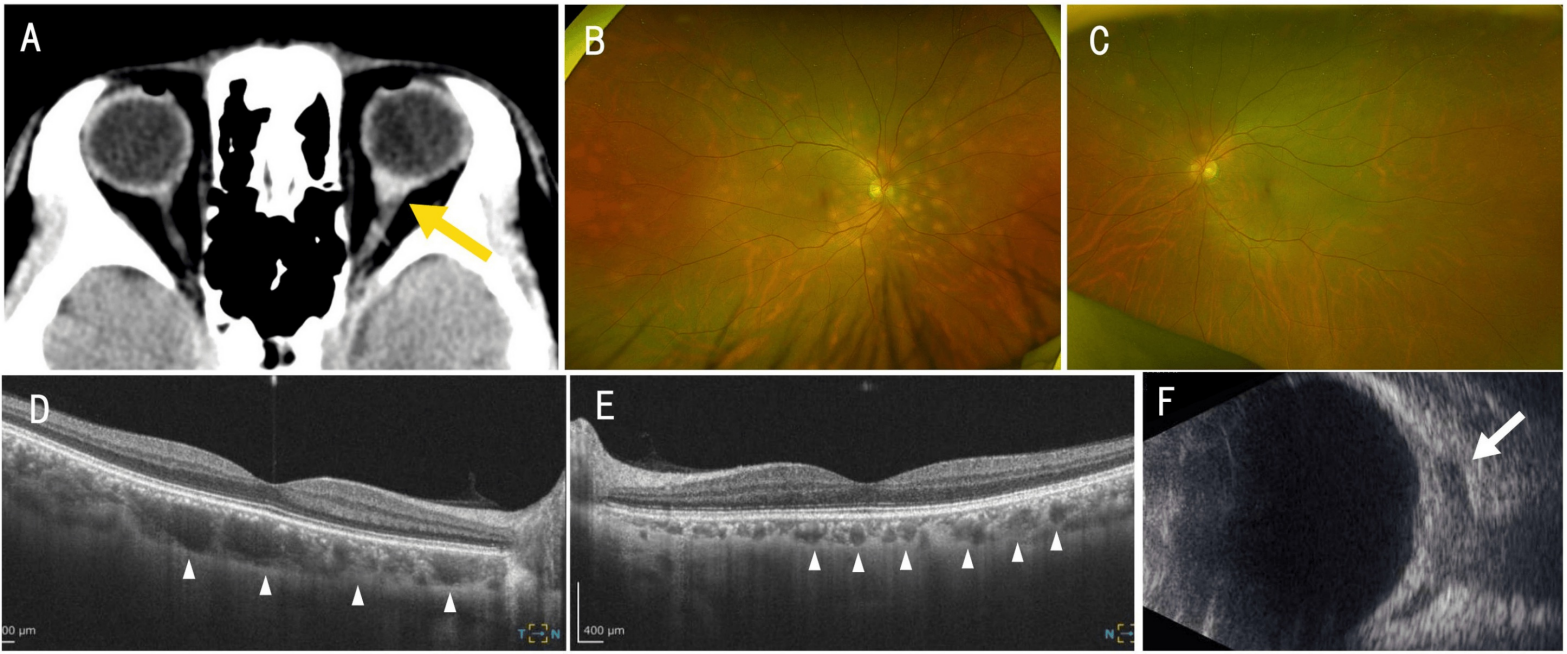
Structural imaging findings of fundus lesions and an optic nerve-associated lesion (A) Non-contrast head computed tomography showing a hyperdense lesion contiguous with the left optic nerve (yellow arrow). (B, C) Ultra-widefield color fundus images of the right eye (B) and the left eye (C), showing multiple deep yellow-orange patchy choroidal lesions. (D, E) Swept-source optical coherence tomography (SS-OCT) images of the right (D) and left (E) eyes show multiple well-defined, hyporeflective, spongiform lesions within the choroidal layer (white arrowheads). Bruch’s membrane, the retinal pigment epithelium, and the neurosensory retina remain intact. (F) B-scan ultrasonography of the left eye also demonstrating a low-echoic lesion continuous with the optic nerve (white arrow).

A 53-year-old man was transported to the emergency department in March 2024 after a motor vehicle collision. Head computed tomography revealed a hyperdense lesion adjacent to the left optic nerve (Figure 1A), and an intraorbital hematoma was suspected. He was referred to the ophthalmology department the following day.

The patient was asymptomatic. Best-corrected visual acuity was 20/16 in the right eye and 20/13 in the left eye, and no relative afferent pupillary defect was observed in either eye. Slit-lamp examination was unremarkable, with no signs suggestive of inflammatory disease, such as anterior chamber cells, keratic precipitates, or vitreous opacities. Fundus examination revealed multiple deep yellow-to-orange patchy choroidal lesions diffusely distributed in the right eye and localized to the superonasal region in the left eye (Figure 1B, 1C). 

Swept-source OCT demonstrated multiple well-demarcated hyporeflective spongiform lesions within the choroid without disruption of Bruch’s membrane, the retinal pigment epithelium, or the neurosensory retina (Figure 1D, 1E). B-scan ultrasonography revealed a hypoechoic lesion contiguous with the optic nerve (Figure 1F).

**Figure 2:**
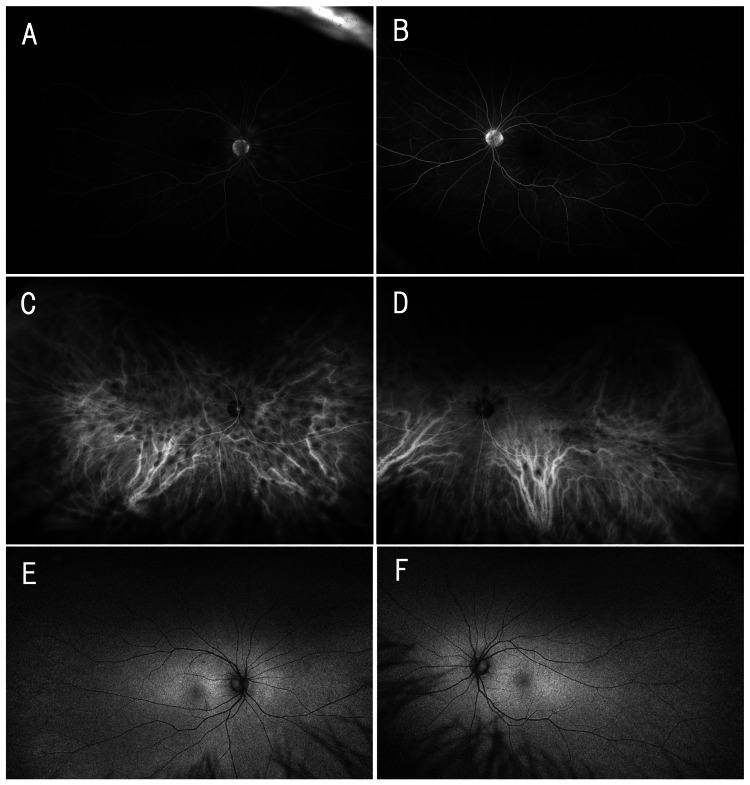
Fundus angiography and short-wavelength fundus autofluorescence findings of fundus lesions (A, B) Late-phase fluorescein angiography (FA) images of the right (A) and left (B) eyes demonstrating staining corresponding to some of the patchy lesions. (C, D) Late-phase indocyanine green angiography (ICGA) images of the right (C) and left (D) eyes reveal multiple hypofluorescent areas, more extensive than those visible on color fundus images or FA images. (E, F) Short-wavelength fundus autofluorescence images of the right (E) and left (F) eyes reveal no areas of hyper- or hypo-autofluorescence. These findings support the absence of retinal pigment epithelium involvement.

FA showed late-phase staining in some lesions (Figure 2A, 2B), whereas ICGA revealed more extensive hypofluorescent areas than those visible on color fundus imaging (Figure 2C, 2D). Short-wavelength fundus autofluorescence showed no definite abnormalities (Figure 2E, 2F).

**Figure 3:**
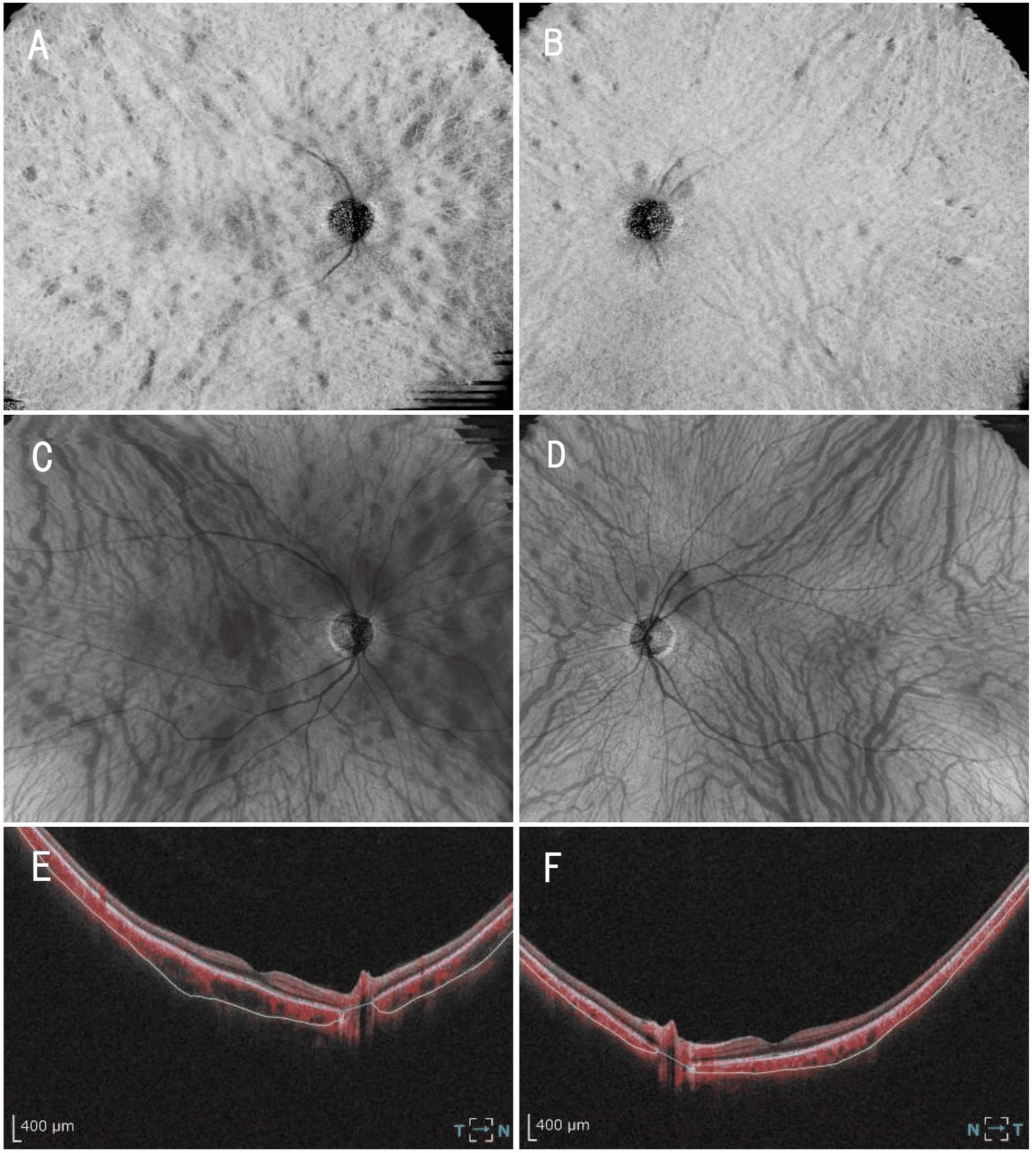
En face OCT and OCT angiography findings at the choroidal level (A, B) En face optical coherence tomography (OCT) images of the choroidal vessels of the right (A) and left (B) eyes depict patchy lesions that are continuous with the underlying choroidal vasculature. (C, D) Choroidal slab OCT angiography (OCTA) images of the right (C) and left (D) eyes demonstrate multiple patchy lesions of variable size. (E, F) B-scan OCTA images of the right (E) and left (F).

Wide-field en face OCT demonstrated patchy lesions continuous with the choroidal vasculature (Figure 3A, 3B). Wide-field OCTA revealed multiple patchy lesions of variable size at the choroidal level, predominantly in the right eye (Figure 3C-3F).

**Figure 4:**
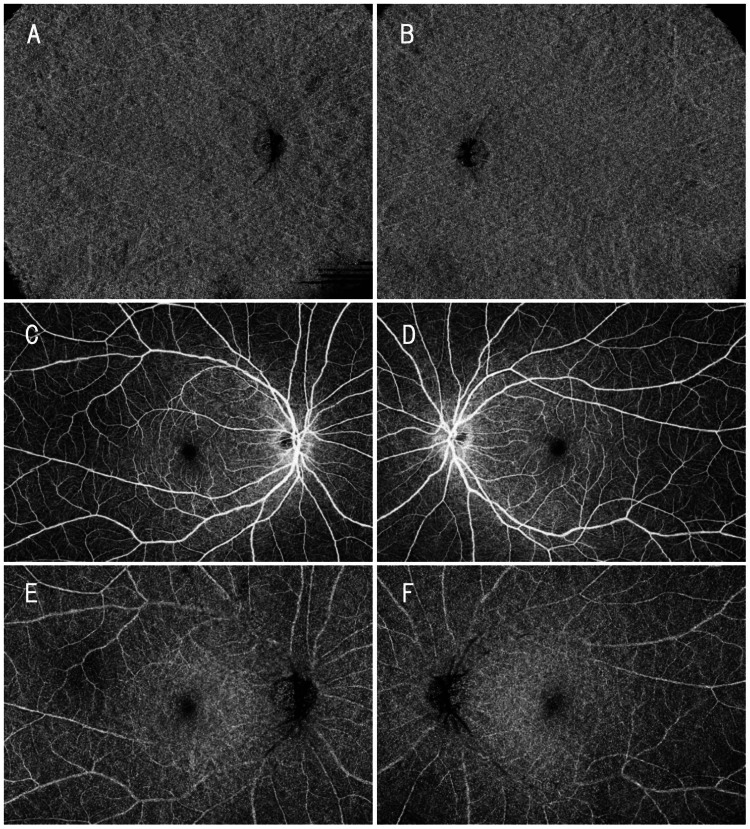
OCT angiography findings at the choriocapillaris and retinal capillary levels (A, B) Choriocapillaris-slab optical coherence tomography angiography (OCTA) images of the right (A) and left (B) eyes demonstrate slight reduction of flow signals corresponding to some of the patchy lesions. (C, D) Superficial retinal capillary plexus slab OCTA images of the right (C) and left (D) eyes. (E, F) Deep retinal capillary plexus slab OCTA images of the right (E) and left (F) eyes. No abnormal findings are observed in either the superficial or deep retinal capillary plexus.

Mild flow signal attenuation corresponding to some lesions was observed in the overlying choriocapillaris (Figure 4A, 4B), while no abnormalities were detected in the superficial or deep retinal capillary plexuses (Figure 4C-4F).

**Figure 5:**
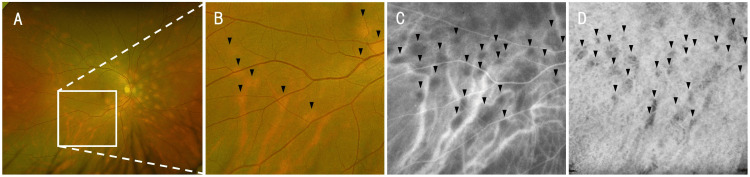
Comparison of choroidal lesions across imaging modalities in the present case The white square region in the Ultra-widefield color fundus image of the right eye (A) is shown at identical locations in the Ultra-widefield color fundus image (B), late-phase indocyanine green angiography (ICGA) (C), and choroidal optical coherence tomography angiography (OCTA) (D). Lesions indicated by black arrowheads are barely visible on the color image but are clearly delineated on ICGA and OCTA. The extent of the lesions on ICGA and OCTA is comparable.

Lesions that were indistinct on color fundus photography were more clearly delineated on indocyanine green angiography and wide-field OCTA, demonstrating a broader extent of involvement (Figure 5).

No visual field abnormalities were detected on static perimetry. Serologic testing performed to exclude infectious etiologies was negative for tuberculosis, syphilis, varicella-zoster virus, toxoplasma, and herpes simplex virus. In addition, whole-body computed tomography and serum tumor markers, including carcinoembryonic antigen (CEA), alpha-fetoprotein (AFP), carbohydrate antigen 19-9 (CA19-9), squamous cell carcinoma antigen (SCC), lactate dehydrogenase (LDH), interleukin-2 receptor (IL-2R), angiotensin-converting enzyme (ACE), and calcium, revealed no abnormalities. Furthermore, evaluation by a hematologist revealed no evidence of systemic disease. Contrast-enhanced magnetic resonance imaging showed a lesion adjacent to the left optic nerve compatible with lymphoma, without other tumorous findings. Biopsy was not performed due to preserved visual acuity, proximity to the optic nerve, and patient preference. Based on multimodal imaging findings, clinical stability, and similarity to previous reports, the lesions were considered most consistent with indolent nonprogressive multifocal choroidal lesions. No progression was observed during one year of follow-up.

## Discussion

This case represents a rare instance of bilateral indolent nonprogressive multifocal choroidal lesions. Given the limited number of reported cases and the absence of established diagnostic criteria, comprehensive evaluation using multimodal imaging plays an important role in clinical assessment and longitudinal monitoring.

In the present case, OCTA demonstrated reduced flow signals at the level of the choroid, whereas the superficial and deep retinal capillary plexuses were preserved. These findings support the concept that the primary site of involvement is the choroid rather than the retina. A previous report described clinicopathologic correlation demonstrating lymphocytic infiltration confined to the outer choroid with sparing of the inner choroid and choriocapillaris [2]. Our findings are consistent with preserved retinal structure; however, we observed mild attenuation of flow signals in the overlying choriocapillaris. Given the subtle nature of this attenuation, it is unlikely to represent direct lymphocytic infiltration and may instead reflect secondary hemodynamic alterations or compression-related vascular compromise.

Multimodal imaging was particularly useful in detecting lesions that were inconspicuous on color fundus photography. Although previous reports have described structural OCT and conventional OCTA findings, detailed evaluation using wide-field imaging modalities has not been well characterized. In this case, wide-field en face OCT enabled simultaneous visualization of multiple lesions, facilitating assessment of lesion distribution and vascular characteristics in a single examination. Compared with ICGA, wide-field OCTA is noninvasive and suitable for repeated follow-up [5]. Because active dye leakage is not a defining feature of this entity, OCTA may be sufficient for monitoring structural and vascular stability.

Most previously reported cases have involved unilateral disease [3,4,6,7]. In contrast, our patient demonstrated bilateral involvement. Although bilateral presentation is uncommon, it has been described in isolated reports [8]. The absence of retinal capillary plexus abnormalities in our case distinguishes it from reports suggesting more extensive inflammatory involvement. Bilaterality may represent either a broader spectrum of the same indolent process or an early manifestation of a systemic lymphoproliferative condition; therefore, careful long-term surveillance is warranted.

The differential diagnosis of multiple white patchy choroidal lesions includes infectious, inflammatory, and neoplastic conditions such as syphilis, tuberculosis, birdshot chorioretinopathy, APMPPE, MEWDS, sarcoidosis, and choroidal lymphoma. Infectious etiologies were excluded in this case based on negative serologic testing for syphilis, tuberculosis, toxoplasmosis, and herpes virus infections.

Birdshot chorioretinopathy is characterized by multiple cream-colored choroidal lesions arranged in a shotgun-like pattern and represents an important differential diagnosis [9]. On FA, it typically shows retinal vasculitis with vascular leakage and irregular venous caliber, as well as cystoid macular edema with petaloid hyperfluorescence [9]. Prolongation of the arteriovenous transit time has also been reported, with previous studies indicating a duration of approximately 31 seconds; in contrast, the transit time in our case was nine seconds, showing no delay [10]. On ICGA, lesions usually appear hypofluorescent in the early to middle phases and become isofluorescent in the late phase, whereas in our case, they were isofluorescent from the early phase [11]. Furthermore, OCT often reveals cystoid macular edema during the disease course, along with choroidal thinning and disruption of the photoreceptor inner segment/outer segment (IS/OS) junction [12]. However, none of these findings were observed in the present case, making birdshot chorioretinopathy unlikely. HLA-A29 testing was not performed, as the clinical and imaging findings made birdshot chorioretinopathy unlikely; however, complete exclusion of this entity is not possible.

Other white dot syndromes, including APMPPE and MEWDS, were also considered. APMPPE typically presents bilaterally with cream-colored placoid lesions in the posterior pole and is associated with acute visual decline, photopsia, and paracentral scotomas. A characteristic feature of FA is fluorescence reversal, with early hypofluorescence followed by late hyperfluorescence. OCT findings include disruption of the outer retinal layers and hyperreflective material at the same level [13]. In contrast, MEWDS is usually unilateral and presents with multiple faint white dots in the posterior pole and mid-periphery, often following a flu-like illness and accompanied by acute visual disturbance, photopsia, and visual field defects. ICGA demonstrates multiple hypofluorescent spots, while fundus autofluorescence shows corresponding hyperautofluorescent lesions. OCT typically reveals disruption of the IS/OS junction [14]. These findings were not consistent with those observed in our case. Sarcoidosis was also considered unlikely due to the absence of systemic involvement, normal serum markers including ACE and IL-2R, and lack of intraocular inflammatory signs.

Differentiation from intraocular lymphoma, particularly choroidal lymphoma, is clinically important. Choroidal lymphoma is generally a low-grade B-cell lymphoma that presents with diffuse or nodular choroidal thickening [15]. On OCT, characteristic undulating or “placid” irregularities of the choroidal surface have been reported [16]. Clinically, the lesions may appear as creamy infiltrative patches and can exhibit a so-called “leopard pattern”-like appearance [17]. These lesions may be associated with subretinal fluid or pigment epithelial detachment and typically show a progressive course [17]. In secondary cases, systemic lymphoproliferative disease and elevated serum markers such as IL-2R may also be observed [18].

In the present case, although choroidal thickening was observed, the characteristic undulating choroidal surface was not evident on OCT. While a partially “leopard pattern”-like appearance was noted on fundus examination, such a pattern is generally attributed to lymphocytic infiltration at the subretinal or sub-RPE level and is often associated with hyperreflective deposits beneath the RPE on OCT [17]. However, no such sub-RPE hyperreflective deposits were identified in this case. In addition, there was no evidence of subretinal fluid or pigment epithelial detachment, and the lesions remained stable without progression over one year of follow-up. Furthermore, systemic evaluation, including serum tumor markers such as IL-2R and whole-body imaging, revealed no abnormalities.

Taken together, the clinical course and imaging findings in this case are not consistent with typical choroidal lymphoma, and its likelihood is considered low. However, a definitive diagnosis requires histopathological confirmation, and since a biopsy was not performed in this case, choroidal lymphoma cannot be completely excluded.

The clinical course in our patient remained stable over one year, supporting the indolent and nonprogressive nature of this condition. However, rare progression to systemic lymphoma has been reported, including a case in which pulmonary low-grade B-cell lymphoma developed 16 years after the initial ocular findings [6]. This observation underscores that, despite its typically benign course, long-term follow-up is essential. If visual deterioration, lesion enlargement, or new systemic findings occur, further systemic evaluation should be considered.

This report is limited by its single-case design. Therefore, caution is warranted in generalizing these imaging characteristics, as lesion distribution and vascular alterations may vary among patients. Larger case series are needed to establish consistent imaging patterns and diagnostic criteria.

## Conclusions

Multimodal imaging, particularly wide-field OCTA and wide-field en face OCT, may be useful for the noninvasive evaluation of choroidal diseases such as indolent nonprogressive multifocal choroidal lesions. These modalities enable detailed assessment of lesion morphology and vascular characteristics while improving the detection of lesions that may be inconspicuous on conventional imaging.

In addition to imaging findings, systemic evaluation and laboratory testing contributed to the exclusion of infectious, inflammatory, and neoplastic conditions, supporting the overall diagnostic assessment. Wide-field OCTA and en face OCT are noninvasive and repeatable, making them well-suited for long-term follow-up, allowing continuous monitoring of disease stability without the need for invasive procedures. Collectively, these imaging approaches may contribute significantly to both clinical assessment and longitudinal management of this rare choroidal condition.
